# Development and validation of a risk stratification model for cardiovascular disease in patients with radiographic axial spondyloarthritis (r-axSpA): a retrospective study

**DOI:** 10.3389/fmed.2026.1852328

**Published:** 2026-06-08

**Authors:** Ting Tan, Minglun Xiao, Liang Zhou, Zhongqun Wen, Xinyi Hu, Yuanjun Zheng, Zeyan Peng

**Affiliations:** Department of Traditional Chinese Medicine Rheumatology, Affiliated Banan Hospital of Chongqing Medical University, Chongqing, China

**Keywords:** alkaline phosphatase, cardiovascular disease, radiographic axial spondyloarthritis, retrospective, risk stratification model

## Abstract

**Background:**

Patients with radiographic axial spondyloarthritis (r-axSpA) face an elevated risk of cardiovascular disease (CVD), yet practical prediction tools remain limited. This study aimed to develop a parsimonious risk stratification model for CVD risk in r-axSpA patients using routine clinical variables.

**Methods:**

This single-center retrospective cross-sectional study enrolled 259 r-axSpA patients (65 with CVD). Least absolute shrinkage and selection operator (LASSO) regression and multivariate logistic regression were used to identify independent predictors. The model's discriminative ability, calibration, and clinical utility were assessed using area under the curve (AUC), calibration plots, and decision curve analysis (DCA).

**Results:**

Four independent predictors were identified: age, hypertension, diabetes mellitus, and alkaline phosphatase (ALP). The model demonstrated excellent discriminative ability in the training set (AUC = 0.888, 95% CI: 0.833–0.942; sensitivity 92.2%; specificity 74.6%) and acceptable performance in the validation set (AUC = 0.741). Calibration was satisfactory (Brier score: 0.115), and DCA confirmed positive net benefit. Subgroup analysis showed robust performance across both sexes.

**Conclusions:**

This study developed a simple yet robust risk stratification tool for CVD in r-axSpA patients using four readily available variables. The model showed good discriminative ability and clinical utility, with ALP emerging as a novel predictor. External validation in prospective cohorts is warranted before clinical implementation.

## Introduction

Radiographic axial spondyloarthritis (r-axSpA) is a chronic inflammatory disease characterized primarily by enthesitis (1). Accumulating evidence indicates that multiple factors—including genetic predisposition, infection, immune-mediated inflammation, and bone metabolic dysregulation—collectively contribute to the onset and progression of r-axSpA (2). Among these, immune system dysfunction is closely associated with chronic inflammation and bone destruction (3–5). r-axSpA predominantly affects young to middle-aged males and is frequently associated with the genetic marker human leukocyte antigen B27 (HLA-B27) (6, 7). The disease has an insidious onset, most commonly involving the sacroiliac joints and spine (8). In severe cases, bone destruction and remodeling may occur, leading to irreversible damage such as spinal deformity and joint ankylosis, which significantly impair patients' quality of life (9). Furthermore, r-axSpA may involve multiple extra-articular organs and systems, including the eyes, cardiovascular system, kidneys, skin and mucous membranes, nervous system, and gastrointestinal tract (10–12).

In recent years, comorbidities associated with spondyloarthritis have attracted increasing attention from researchers worldwide. Cardiovascular disease (CVD) is a common comorbidity in spondyloarthritis, including r-axSpA and psoriatic arthritis (13, 14). In patients with r-axSpA, cardiovascular manifestations are often insidious and variable, potentially involving the blood vessels, cardiac conduction system, myocardium, and pericardium (15). Among these, heart failure and stroke are relatively prevalent and associated with high mortality, significantly reducing life expectancy and posing major challenges in the clinical management of r-axSpA (16). A longitudinal study involving 133 r-axSpA patients reported a total CVD prevalence of 13.5%, which is markedly higher than that in the general population (17). Another study from Spain demonstrated that both clinical and subclinical cardiovascular abnormalities are frequent in r-axSpA patients and are correlated with higher disease activity (18).

Therefore, this study aimed to develop a risk assessment model based on electronic health records to estimate the risk of CVD in patients with r-axSpA. This model is intended to provide clinicians with a practical and user-friendly tool for identifying r-axSpA patients at high risk of developing CVD, using routinely available demographic data, vital signs, and laboratory measurements without the need for specialized equipment.

## Methods

### Data source and study design

This study was a retrospective cross-sectional observational study. Data were obtained from the electronic medical record system of Affiliated Banan Hospital of Chongqing Medical University, and patients with r-axSpA admitted between January 2019 and December 2025 were enrolled. All predictor and outcome variables were collected from historical records at a single time point (the first hospitalization encounter), without longitudinal follow-up for incident CVD events. Therefore, this design supports associational prediction rather than causal inference. The study protocol was approved by the Ethics Committee of the Affiliated Banan Hospital of Chongqing Medical University (approval number: BNLL-KY-2025-002-1). Because the analysis was based solely on anonymized historical data without any patient intervention, the requirement for informed consent was waived by the Ethics Committee.

**Figure 1:**
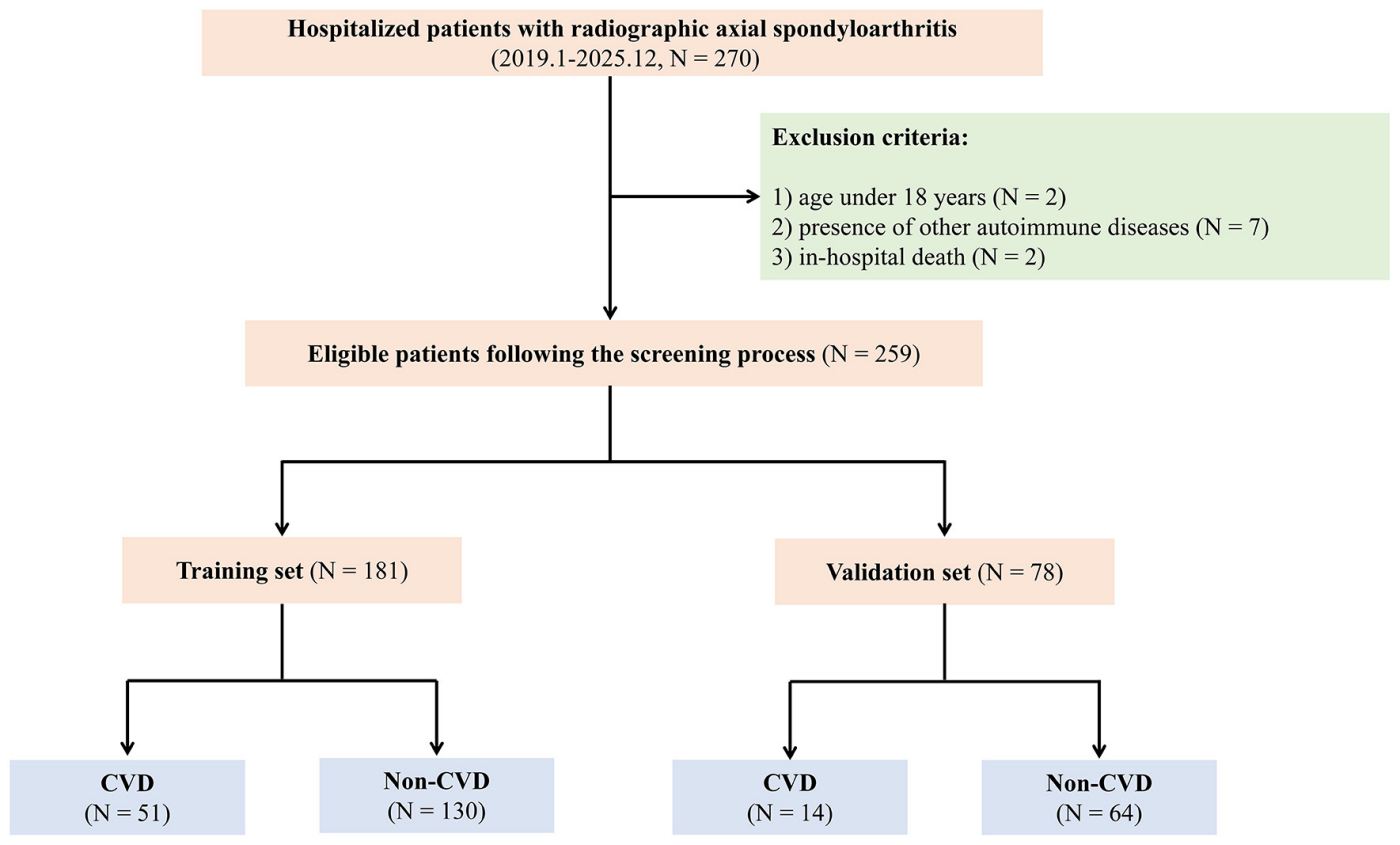
Flowchart of the study.

The inclusion criteria were as follows: (1) hospitalized patients with a confirmed diagnosis of r-axSpA; and (2) hospital stay exceeding 48 h. The exclusion criteria were: (1) age under 18 years; (2) presence of other autoimmune diseases, specifically systemic lupus erythematosus, Sjögren‘s syndrome, and psoriasis; and (3) in-hospital death. All eligible patients were randomly divided into a training set (for model development) and a validation set (for internal validation) at a ratio of 7:3 using stratified random sampling. The workflow of the study is illustrated in Figure 1.

### Diagnostic criteria

Diagnosis of r-axSpA: According to the modified New York criteria of 1984, patients must present with radiographic sacroiliitis of bilateral grade ≥2 or unilateral grade ≥3, along with at least one of the following clinical criteria: (1) low back pain and morning stiffness lasting for ≥3 months and improving with activity; (2) limited motion of the lumbar spine in both sagittal and frontal planes; and (3) reduced chest expansion relative to normal values for age and gender (19). For atypical cases, the diagnosis was confirmed by two or more attending physicians or senior specialists in rheumatology and immunology.

Definition of CVD: In this study, CVD was defined as the presence of any of the following events: coronary artery disease (including coronary atherosclerotic heart disease, post-coronary stent implantation status, and angina pectoris); cerebrovascular and atherosclerotic diseases (cerebral arterial insufficiency, cerebral artery stenosis, internal carotid atherosclerosis, carotid atherosclerosis, cerebral atherosclerosis); or heart failure and arrhythmias (cardiac function grade ≥II, chronic heart failure, arrhythmia, and chronic cor pulmonale). The inclusion of arrhythmias and chronic cor pulmonale is justified by well-established evidence that r-axSpA patients have an increased risk of cardiac conduction system abnormalities (due to fibrosis and inflammation of the conducting tissue) and pulmonary involvement (secondary to chest wall restriction, costovertebral joint involvement, and chronic inflammation). Although these conditions differ from classical atherosclerotic CVD, they represent clinically important cardiovascular complications in r-axSpA patients that significantly impact prognosis and quality of life, and their inclusion in a composite endpoint for risk stratification is clinically meaningful.

### Data collection and data quality control

The predictors included in this study cover multiple clinical dimensions. Demographic characteristics consist of age and gender. Medical history involves hypertension, diabetes mellitus (DM), hyperlipidemia, and chronic liver disease (CLD). Hemodynamic indicators include pulse pressure, systolic blood pressure (SBP), and diastolic blood pressure (DBP). Lipid metabolism indicators include total cholesterol (TC), triglycerides (TG), low-density lipoprotein cholesterol (LDL-C), and high-density lipoprotein cholesterol (HDL-C). Coagulation function parameters comprise international normalized ratio (INR), activated partial thromboplastin time (APTT), and D-dimer. Inflammatory and immune-related indicators include systemic immune-inflammation index (SII), neutrophil percentage-to-albumin ratio (NPAR), neutrophil-to-lymphocyte ratio (NLR), platelet-to-lymphocyte ratio (PLR), and lymphocyte-to-monocyte ratio (LMR). Liver and kidney function along with metabolic indicators include serum creatinine (Crea), uric acid (UA), alkaline phosphatase (ALP), gamma-glutamyl transferase (GGT), and total bilirubin (TBIL). Electrolyte parameters consist of potassium (K^+^), sodium (Na^+^), chloride (Cl^−^), calcium (Ca^2+^), and phosphorus (P).

Given that multiple laboratory measurements might be available for the same patient during hospitalization, only the first test result after admission was selected for statistical analysis in this study. Considering the extended study period, the same laboratory parameter might have been recorded in different units; therefore, all units were standardized. Furthermore, variables with a missing rate exceeding 30% were excluded, whereas those with a missing rate below 30% were handled using multiple imputation.

### Statistical analysis

Data processing and statistical analyses were performed using R software (version 4.3.3) and SPSS 26.0. All tests were two-sided, and a *P* value < 0.05 was considered statistically significant. Baseline characteristics of patients in the training and validation sets were described. Continuous variables following a normal distribution were expressed as mean ± standard deviation, with intergroup comparisons conducted using the independent samples *t*-test. For those not following a normal distribution, the median (interquartile range) was reported, and the Mann–Whitney U test was used for between-group comparisons. Categorical variables were presented as frequencies (percentages), and group differences were evaluated using the χ^2^ test or Fisher's exact test, as appropriate.

To minimize overfitting and identify optimal predictors, least absolute shrinkage and selection operator (LASSO) regression was employed for variable dimensionality reduction. The optimal penalty coefficient λ was determined via ten-fold cross-validation following the 1-SE criterion. Variables with non-zero coefficients selected by LASSO were subsequently incorporated into a multivariate binary logistic regression model to establish a risk stratification model for CVD in r-axSpA patients. Regression results were reported as odds ratios (ORs) with corresponding 95% confidence intervals (CIs). Discriminative ability was assessed using the area under the receiver operating characteristic curve (AUC) (22). Calibration was evaluated using calibration curves supplemented by the Hosmer–Lemeshow goodness-of-fit test (23). Clinical net benefit was quantified by decision curve analysis (DCA) across various threshold probabilities (24). Additionally, sensitivity, specificity, positive likelihood ratio (PLR), negative likelihood ratio (NLR), and diagnostic odds ratio (DOR) were calculated. All metrics were computed separately for the training and validation sets to assess model stability.

The dose–response relationship between continuous variables (e.g., age, APTT, ALP) and CVD risk was explored using restricted cubic spline (RCS) regression with 3 to 5 knots, where the optimal number of knots was determined based on the Akaike information criterion (25). Nonlinearity was tested, and the corresponding *P* value for nonlinearity was reported. Furthermore, subgroup analyses were also performed in this study.

## Results

### Study population and baseline characteristics

A total of 259 patients with r-axSpA who met the inclusion and exclusion criteria were ultimately enrolled in this study, of whom 65 had CVD, accounting for 25.10% of the total. Participants were randomly divided into a training set (*n* = 181) and a validation set (*n* = 78) at a ratio of 7:3. In the training set, there were 51 cases in the CVD group and 130 cases in the non-CVD group; in the validation set, there were 14 cases in the CVD group and 64 cases in the non-CVD group. In both the training and validation sets, significant differences were observed only in age and TG levels (*P* < 0.05), indicating reasonable overall partitioning (Table 1). The imputation results for indicators with a missing rate of less than 30% in the training and validation sets are detailed in Supplementary Tables 1, 2, respectively, and showed no statistically significant differences before and after imputation (*P* > 0.05) in either dataset.

**Table 1:** Baseline characteristics of r-axSpA patients in the training and validation sets.

Variables	Total (*n* = 259)	Training set (*n* = 181)	Validation set (*n* = 78)	*P* value
Age (years)	46 (32.5, 60.5)	48 (34, 62)	37.5 (30, 55.75)	0.013
Gender, *n* (%)	
Male	173 (66.8)	123 (67.96)	50 (64.1)	0.645
Female	86 (33.2)	58 (32.04)	28 (35.9)	
Hypertension, *n* (%)	
Yes	56 (21.62)	42 (23.2)	14 (17.95)	0.437
No	203 (78.38)	139 (76.8)	64 (82.05)	
DM, *n* (%)	
Yes	30 (11.58)	23 (12.71)	7 (8.97)	0.516
No	229 (88.42)	158 (87.29)	71 (91.03)	
Hyperlipidemia, *n* (%)	
Yes	32 (12.36)	23 (12.71)	9 (11.54)	0.955
No	227 (87.64)	158 (87.29)	69 (88.46)	
CLD, *n* (%)	
Yes	43 (16.6)	30 (16.57)	13 (16.67)	1.000
No	216 (83.4)	151 (83.43)	65 (83.33)	
Pulse pressure (mmHg)	82 (75, 90.5)	82 (74, 90)	82 (76, 91.5)	0.684
SBP (mmHg)	123 (110, 136)	124 (112, 138)	120 (109, 129.5)	0.098
DBP (mmHg)	78 (70, 86)	78 (71, 87)	78 (70, 83)	0.422
TC (mmol/L)	3.9 (3.57, 4.64)	4 (3.57, 4.78)	3.76 (3.53, 4.28)	0.059
TG (mmol/L)	0.99 (0.71, 1.43)	1.06 (0.8, 1.46)	0.91 (0.68, 1.37)	0.017
LDL-C (mmol/L)	2.61 (2.01, 3.15)	2.7 (1.98, 3.24)	2.35 (2.07, 2.91)	0.278
HDL-C (mmol/L)	1.11 (0.92, 1.33)	1.11 (0.91, 1.35)	1.11 (0.94, 1.22)	0.602
INR	1 (0.94, 1.07)	1 (0.94, 1.07)	1.01 (0.92, 1.08)	0.916
APTT (s)	31.6 (28.25, 35.3)	31.7 (27.9, 35.6)	31.35 (28.93, 34.88)	0.979
D-dimer (mg/L)	0.28 (0.22, 0.52)	0.29 (0.23, 0.51)	0.26 (0.19, 0.61)	0.121
SII	694.8 (426.11, 1,300)	698.4 (426.01, 1,389.62)	694.8 (428.96, 985.99)	0.264
NPAR	16.36 (13.88, 19.16)	16.58 (14.05, 19.42)	15.82 (13.65, 18.71)	0.314
NLR	2.94 (1.94, 5.42)	3.06 (1.98, 5.81)	2.69 (1.9, 4.55)	0.115
PLR	158.16 (119.17, 221.01)	152.38 (121.16, 217.02)	178.99 (117.8, 224.17)	0.497
LMR	3.89 (2.41, 5.32)	3.86 (2.31, 5.21)	4.05 (2.97, 5.54)	0.160
Crea (μmol/L)	62 (52, 71)	62 (52, 71)	59 (52.25, 71)	0.707
UA (μmol/L)	341 (274.5, 407.5)	340 (276, 399)	347 (274.25, 415)	0.996
ALP (U/L)	81 (66.5, 102.5)	80 (66, 101)	81 (68, 105.75)	0.677
GGT (U/L)	22 (14.5, 38.5)	22 (15, 20)	20 (14, 21)	0.405
TBIL (μmol/L)	11.1 (8.65, 15)	11 (8.7, 15.7)	11.2 (8.65, 14.9)	0.959
K^+^ (mmol/L)	3.92 (3.68, 4.18)	3.94 (3.68, 4.16)	3.92 (3.68, 4.2)	0.612
Na^+^ (mmol/L)	140 (138.75, 141.9)	140 (138.8, 142)	140.1 (138.75, 141.23)	0.884
Cl^−^ (mmol/L)	105 (103, 107)	105 (102.4, 107)	105 (103.25, 107)	0.496
Ca^2+^ (mmol/L)	2.27 (2.2, 2.35)	2.26 (2.18, 2.35)	2.28 (2.23, 2.37)	0.092
P (mmol/L)	1.06 (0.97, 1.21)	1.05 (0.97, 1.2)	1.07 (0.94, 1.24)	0.647

DM, diabetes mellitus; CLD, chronic liver disease; SBP, systolic blood pressure; DBP, diastolic blood pressure; TC, total cholesterol; TG, triglycerides; LDL-C, low-density lipoprotein cholesterol; HDL-C, high-density lipoprotein cholesterol; INR, international normalized ratio; APTT, activated partial thromboplastin time; SII, systemic immune-inflammation index; NPAR, neutrophil percentage-to-albumin ratio; NLR, neutrophil-to-lymphocyte ratio; PLR, platelet-to-lymphocyte ratio; LMR, lymphocyte-to-monocyte ratio; Crea, creatinine; UA, uric acid; ALP, alkaline phosphatase; GGT, gamma-glutamyl transferase; TBIL, total bilirubin; K^+^, potassium; Na^+^, sodium; Cl^−^, chloride; Ca^2+^, calcium; P, phosphorus.

### Variable selection and independent predictors

**Figure 2:**
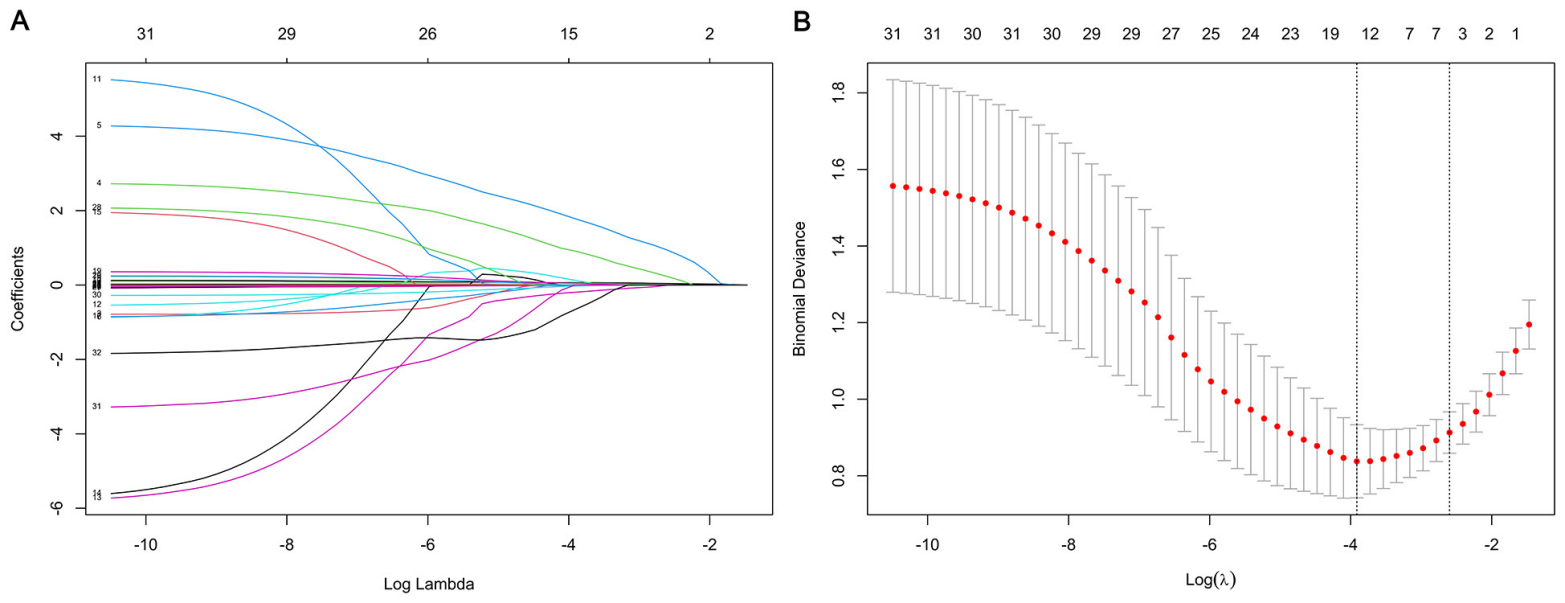
Variable selection using LASSO regression. **(A)** Cross-validation curve for tuning parameter λ selection; **(B)** LASSO coefficient profiles of 31 candidate variables.

In the training set, all 31 candidate predictors were included in the LASSO regression analysis. At the optimal lambda value of lambda.1se = 0.07445363, five variables with non-zero coefficients were identified: age, hypertension, DM, APTT, and ALP (Figure 2). Subsequent multivariate logistic regression analysis revealed that age (OR = 1.088, 95% CI: 1.059–1.123, *P* < 0.001), hypertension (OR = 3.589, 95% CI: 1.297–10.322, *P* < 0.001), DM (OR = 14.672, 95% CI: 3.553–74.510, *P* < 0.001), APTT (OR = 1.129, 95% CI: 1.039–1.242, *P* < 0.001), and ALP (OR = 0.982, 95% CI: 0.966–0.991, *P* < 0.001) were independently associated with CVD.

### Dose-response relationships of continuous predictors

**Figure 3:**
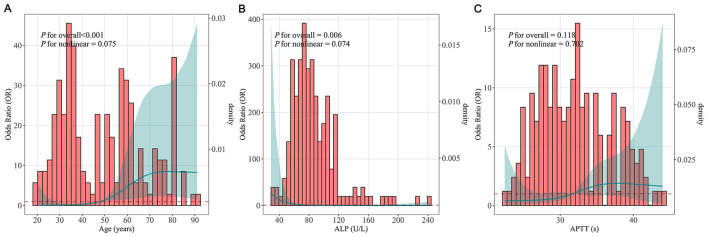
Dose-response relationships of continuous predictors using restricted cubic spline (RCS) analysis. **(A)** Age; **(B)** ALP; **(C)** APTT.

RCS analysis revealed distinct association patterns with CVD outcomes. Age demonstrated a significant overall association (*P* for overall <0.001) with a monotonic increase in risk, while the test for nonlinearity was not significant (*P* for nonlinear = 0.075) (Figure 3A). ALP showed a significant overall association (*P* for overall = 0.006) with a U-shaped pattern, indicating elevated risk at both low and high levels, and a borderline nonlinear trend (*P* for nonlinear = 0.074) (Figure 3B). Thus, the linear model (OR = 0.982) captured a net negative association because the majority of observations lie in the mid-range of the ALP distribution, whereas the RCS analysis reveals that risk is elevated at both extremes. Because the nonlinear trend did not reach statistical significance and the ascending limb of the U-shaped curve was based on limited observations, ALP was entered as a linear term in the final logistic regression model to maintain parsimony and avoid overfitting. In contrast, APTT was not significantly associated with the outcome (*P* for overall = 0.118), with no evidence of nonlinearity (*P* for nonlinear = 0.702) (Figure 3C).

### Construction of the nomogram prediction model

**Figure 4:**
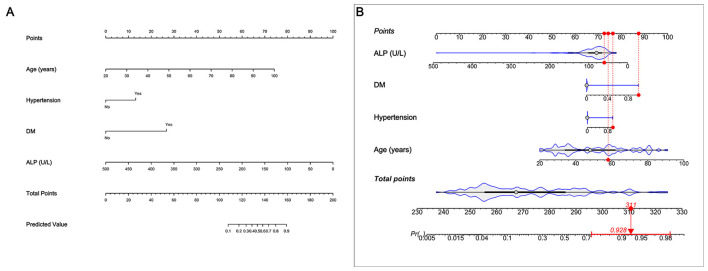
Nomogram for predicting CVD risk in r-axSpA patients. **(A)** Nomogram; **(B)** Representative case illustration (a 58-year-old patient).

Based on the dose-response relationship analyses described above, we further evaluated the incremental predictive value of APTT. Specifically, we compared models with and without APTT (both built upon age, hypertension, DM, and ALP), and found no significant improvement with APTT (DeLong test, *P* = 0.083). We then evaluated the incremental predictive value of ALP by comparing a base model including conventional cardiovascular predictors (age, hypertension, and DM) versus an extended model additionally incorporating ALP. The extended model showed improved discrimination (AUC: 0.888 vs. 0.845; DeLong test, *P* = 0.041) and better reclassification (NRI = 0.312, *P* = 0.002; IDI = 0.058, *P* = 0.002), with lower AIC and BIC values indicating superior model fit. Detailed results are presented in Supplementary Table 3. Accordingly, the four-predictor model (age, hypertension, DM, and ALP) was selected as the final model. A nomogram visualizing the final model is presented in Figure 4A. Figure 4B illustrates a representative case: a 58-year-old r-axSpA patient with comorbid hypertension and DM and an ALP level of 60 U/L, yielding a predicted probability of CVD as high as 92.8%.

### Evaluation and validation of the prediction model

**Figure 5:**
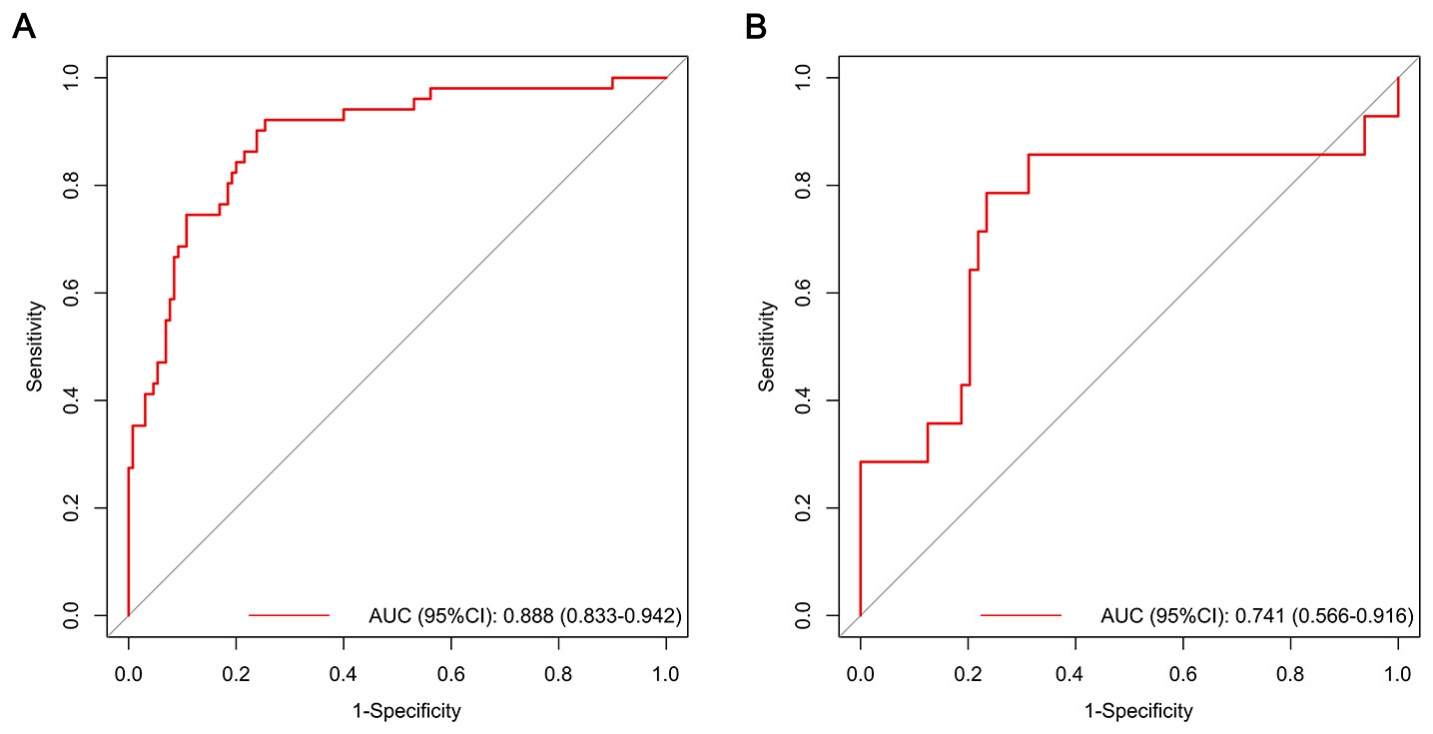
Receiver operating characteristic (ROC) curves of the risk stratification model. **(A)** Training set; **(B)** Validation set.

**Figure 6:**
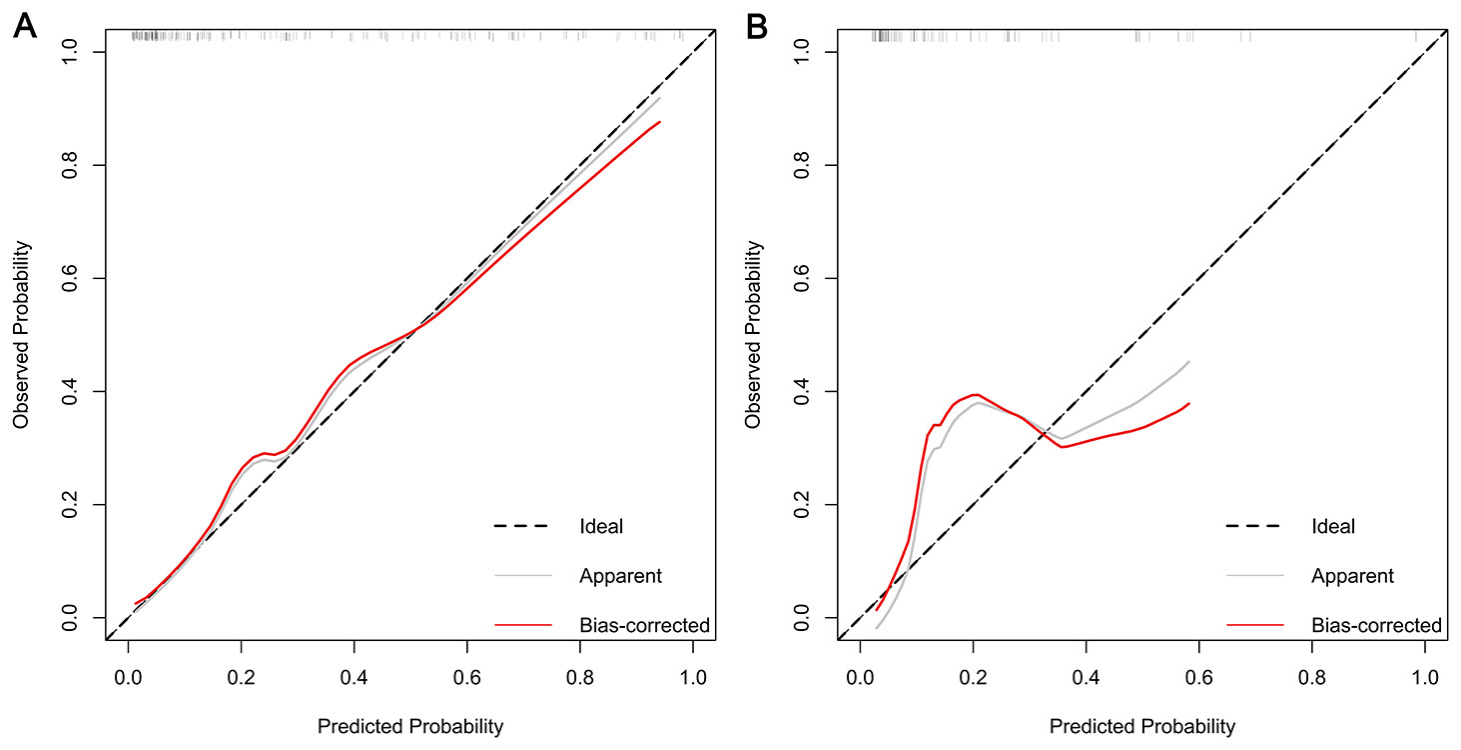
Calibration curves of the risk stratification model. **(A)** Training set; **(B)** Validation set.

**Figure 7:**
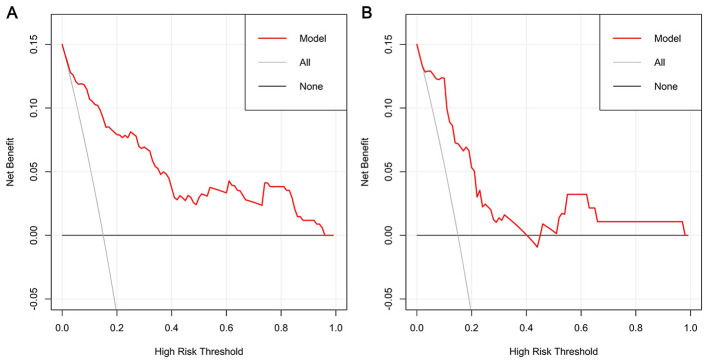
Decision curve analysis (DCA) of the risk stratification model. **(A)** Training set; **(B)** Validation set.

Following nomogram construction, the model demonstrated excellent discriminative ability, with AUCs of 0.888 (95% CI: 0.833–0.942) in the training set and 0.741 (95% CI: 0.566–0.916) in the validation set (Figure 5). The corresponding sensitivities were 92.2% and 78.6%, and specificities were 74.6% and 76.6%, respectively. Calibration was satisfactory, with Brier scores of 0.115 and 0.130 in the training and validation sets, respectively (Figures 6C, D), and DCA confirmed positive net benefit across a range of risk thresholds (Figure 7). Additional performance metrics, including PLR, NLR and DOR, are summarized in Table 2.

**Table 2:** Additional performance metrics of the risk stratification model.

Performance indicators	Training set	Validation set
AUC (95% CI)	0.888 (0.833–0.942)	0.741 (0.566-0.916)
Sensitivity (95% CI)	0.922 (0.848–0.995)	0.786 (0.571–1.000)
Specificity (95% CI)	0.746 (0.671–0.821)	0.766 (0.662–0.869)
PLR (95% CI)	3.630 (2.675–4.927)	3.352 (1.992–5.642)
NLR (95% CI)	0.105 (0.041–0.271)	0.280 (0.102–0.770)
DOR (95% CI)	34.538 (11.559–103.20)	11.978 (2.949–48.646)

AUC, area under the ROC curve; PLR, positive likelihood ratio; NLR, negative likelihood ratio; DOR, diagnostic odds ratio; CI, confidence interval.

### Subgroup analysis

To further evaluate the stability of the model, a subgroup analysis stratified by gender was conducted within the training set. In the male subgroup, the model achieved an AUC of 0.870 (95% CI: 0.779–0.962), with a sensitivity of 94.7% and specificity of 71.8%. In the female subgroup, the AUC was 0.894 (95% CI: 0.824–0.964), with a sensitivity of 84.4% and specificity of 83.5%. The results remained stable across subgroups, indicating satisfactory robustness of the model. Additional performance metrics are detailed in Supplementary Table 4.

## Discussion

Accumulating evidence has demonstrated that patients with r-axSpA are at an increased risk of CVD (26, 27). It has been reported that cardiovascular involvement occurs in approximately 2% to 10% of r-axSpA patients, and its prevalence is closely associated with disease duration (28, 29). A meta-analysis conducted by Sylvain Mathieu and colleagues, which included 11 longitudinal studies, assessed the risk of cerebrovascular and cardiovascular events in patients with r-axSpA (30). The results revealed that, compared with healthy controls, r-axSpA patients had a significantly higher risk of myocardial infarction and stroke, with relative risks (RRs) of 1.44 (95% CI: 1.25–1.67) and 1.37 (95% CI: 1.08–1.73), respectively. These findings were further corroborated by a prospective study involving 738 r-axSpA patients, which reported a significantly higher 5-year incidence of CVD in r-axSpA patients compared to the non-inflammatory arthritis population (HR = 4.6, *P* = 0.02) (31). Therefore, developing a robust risk stratification model for CVD in r-axSpA patients is of great clinical significance, as it enables early risk stratification and facilitates individualized prevention strategies. Imaging studies have confirmed subclinical atherosclerosis in inflammatory arthritides, with increased carotid intima-media thickness reported in both psoriatic arthritis and axial spondyloarthritis, providing direct pathophysiological evidence supporting our model (32, 33).

Given the discrepancy between LASSO selection and RCS analysis, we further compared the predictive performance of models with and without APTT. The results showed that excluding APTT did not significantly degrade model performance (DeLong test, *P* > 0.05). This finding suggests that although APTT was selected by LASSO—potentially due to capturing conditional associations or residual confounding—it did not provide independent incremental predictive value, nor did RCS analysis reveal a significant marginal association with the outcome. Given the lack of a clear biological rationale for APTT in atherosclerotic CVD, the final model excluded APTT and retained four predictors: age, hypertension, DM, and ALP. This parsimonious set of variables balances statistical efficiency with enhanced clinical interpretability. The final model demonstrated excellent predictive performance, with an AUC of 0.888 (95% CI: 0.833–0.942), a sensitivity of 92.2%, and a specificity of 74.6%, indicating its potential utility as a practical tool for cardiovascular risk stratification in patients with r-axSpA.

Epidemiological data indicate that the standardized incidence ratios (SMRs) for hypertension and DM in patients with r-axSpA are 1.98 (95% CI: 1.72–2.28) and 1.41 (95% CI: 1.10–1.78), respectively, both significantly higher than those observed in the general population (34). Notably, Vinker Shuster et al. conducted a large-scale cross-sectional study involving 4,076 r-axSpA patients and found that the proportions of traditional cardiovascular risk factors, including hypertension, DM, and dyslipidemia, were significantly higher in r-axSpA patients than in age- and sex-matched controls (35). However, after multivariable adjustment, r-axSpA itself was not identified as an independent risk factor for ischemic heart disease. This finding suggests that the clustering of traditional cardiovascular risk factors, rather than r-axSpA *per se*, may be the predominant driver of elevated cardiovascular risk in this population. From a pathophysiological perspective, chronic systemic inflammation associated with AS may induce endothelial dysfunction, promote insulin resistance, and activate the renin–angiotensin system, thereby accelerating the development and progression of hypertension and diabetes, and forming a vicious cycle involving inflammation, metabolism, and vascular injury (36–38). Therefore, the inclusion of age, hypertension, and DM in the present model essentially captures the “traditional factor” dimension of cardiovascular risk in patients with r-axSpA.

As a unique predictor in the present model, ALP warrants further investigation regarding its association with CVD risk in patients with r-axSpA. Existing evidence indicates that patients with r-axSpA exhibit characteristic alterations in lipid metabolism, including a positive correlation between serum triglyceride levels and disease activity, along with a paradoxical decrease in TC levels (39). This inflammation-related dyslipidemia may lead to functional impairment of HDL-C particles, thereby attenuating their anti-atherosclerotic capacity. ALP, an enzyme associated with bone metabolism and hepatobiliary function, may serve as a marker of systemic inflammatory burden and ectopic calcification tendency in r-axSpA patients when its levels are elevated (20, 40). Previous studies have confirmed that involvement of the aortic valve and conduction system in r-axSpA is closely related to disease duration, and ALP may participate in this process as a surrogate marker of vascular calcification (21, 41). Although ALP has not received sufficient attention in traditional cardiovascular risk stratification, the present study suggests that it holds independent predictive value in the r-axSpA population.

Several limitations should be acknowledged. First, this was a single-center retrospective cross-sectional study, which inherently limits causal inference. Because predictors and the CVD outcome were ascertained contemporaneously from historical records, the temporal sequence cannot be definitively established. Consequently, the associations reported herein should be interpreted as risk stratification rather than causal effects. Second, the retrospective nature may have introduced selection bias (only hospitalized r-axSpA patients) and information bias (laboratory measurements not uniformly timed). Several relevant confounders (BASDAI, inflammatory markers, smoking status, biologic agents, NSAIDs, corticosteroids) were not adequately captured due to lack of routine documentation (BASDAI), single-timepoint measurement (inflammatory markers), high missing rates (smoking status >30%), or indication bias (treatment exposures). Consequently, residual confounding cannot be entirely excluded. Third, the validation cohort was small (78 patients, only 14 CVD events), making performance estimates inherently unstable, as reflected by the wide 95% CI for AUC (0.566–0.916). The observed decrease in AUC from training (0.888) to validation (0.741) likely reflects a combination of overfitting and instability in a small sample. Larger external validation cohorts are urgently needed. Fourth, the association between ALP and CVD warrants cautious interpretation. Logistic regression suggested a linear inverse association (OR = 0.982), while RCS revealed a U-shaped pattern with borderline significance (*P* = 0.074). ALP was modeled as a linear term because the nonlinear trend did not reach significance and sample size was insufficient for complex nonlinear functions. Larger studies are needed to validate the true functional form of ALP. Fifth, disease duration was not available due to insidious onset and inconsistent documentation, representing a potential source of residual confounding. Sixth, the composite CVD endpoint included arrhythmias and chronic cor pulmonale, which have distinct pathophysiologies from atherosclerotic CVD but are clinically relevant complications in r-axSpA. The small event number precluded separate analyses by CVD subtype. The model has not been externally validated; prospective cohort studies are needed to confirm its accuracy and stability.

## Conclusions

In conclusion, this study developed a parsimonious risk stratification model for CVD in patients with r-axSpA using four readily available variables: age, hypertension, diabetes mellitus, and ALP. The model demonstrated excellent discriminative ability (AUC = 0.888) and good clinical utility. Notably, ALP emerged as a novel predictor, reflecting systemic inflammatory burden and ectopic calcification. External validation in prospective cohorts is warranted before clinical implementation.

## Data Availability

The raw data supporting the conclusions of this article will be made available by the authors, without undue reservation.
